# Emerging Roles of C-C Motif Ligand 11 (CCL11) in Cancers and Liver Diseases: Mechanisms and Therapeutic Implications

**DOI:** 10.3390/ijms26104662

**Published:** 2025-05-13

**Authors:** Jiaqi Wang, Kwan Man, Kevin Tak-Pan Ng

**Affiliations:** Department of Surgery, HKU-SZH & School of Clinical Medicine, Li Ka Shing Faculty of Medicine, The University of Hong Kong, Hong Kong, China; wangjq08@connect.hku.hk

**Keywords:** chemokine CCL11, inflammation, cancer, liver disease, therapeutic target

## Abstract

C-C motif ligand 11 (CCL11) is a multifunctional chemokine that regulates immunity, angiogenesis, and tissue remodeling. In addition to its allergic inflammation role, CCL11 exhibits context-dependent dual functions in relation to cancer progression. In liver diseases, it mediates injury, fibrosis, and inflammation while serving as a disease biomarker. This review systematically examines CCL11–receptor interactions and their immunomodulatory mechanisms in cancers and hepatic pathologies. We highlight CCL11’s therapeutic potential as both a prognostic marker and immunotherapeutic target. By integrating molecular and clinical insights, this work advances the understanding of CCL11’s pathophysiological roles and facilitates targeted therapy development.

## 1. Introduction

### 1.1. Genetic, Molecular, and Protein Structure

The C-C motif ligand 11 (CCL11), which was initially identified as eotaxin-1, was first characterized by Williams et al. in 1994 through their investigation of allergic airway inflammation in guinea pigs [1]. As a member of the CC chemokine family, CCL11 exhibits significant structural homology with the macrophage chemoattractant protein (MCP) subfamily. The eotaxin and MCP genes are co-localized on chromosome 17q11, which is a genomic region that also encodes multiple CC chemokines, including MIP-1, I-309, RANTES, and HCC-1,2. Subsequent studies have identified two additional eosinophil-specific CC chemokines—CCL24 (eotaxin-2) and CCL26 (eotaxin-3)—which complete the eotaxin family triad [2].

CCL11, which is a key mediator in the selective recruitment of eosinophils to inflammatory sites during allergic responses, has been extensively investigated in asthma and related eosinophil-associated pathologies [3]. Notably, elevated eotaxin-1 expression levels have been detected across diverse pathological conditions, encompassing both inflammatory diseases and malignancies. Accumulating evidence demonstrates that eotaxin-1 modulates multiple target cells through complex signaling networks [4].

### 1.2. Secretion Resources

CCL11 displays tissue-specific expression patterns, with particularly high levels being observed in the heart, kidney, small intestine, colon, and pancreas, while there is lower expression in the liver, lung, ovary, and placenta. This chemokine is synthesized by diverse cell types, including eosinophils, fibroblasts, endothelial cells, macrophages, B lymphocytes, and chondrocytes. Its expression is tightly regulated by cytokine networks, being upregulated by Th2-associated cytokines (IL-4, IL-10, IL-13, and IL-17) and downregulated by Th1 cytokines such as IFN-γ. The CCL11 promoter region contains critical regulatory elements, including STAT6 and NF-κB binding sites, along with response elements for IFN-α and glucocorticoids [5]. A comprehensive summary of CCL11 production and secretion sources is provided in Table 1.

#### 1.2.1. Eosinophils

In various inflammatory pathologies, including asthma, ulcerative colitis, Crohn’s disease, and drug-induced liver injury, CCL11 production is significantly upregulated in eosinophils, particularly in circulating eosinophils [6]. This production is predominantly stimulated by Th2 cytokines, with IL-4 being a key regulator. Importantly, eosinophil-derived CCL11 establishes an autocrine signaling loop, directly promoting eosinophil chemotaxis, therefore contributing to the pathogenesis of these inflammatory disorders [7].

#### 1.2.2. Fibroblasts

In hepatic tissues, hepatic stellate cells (HSCs) serve as the principal source of CCL11, driving liver fibrogenesis through the recruitment of immune cells, particularly eosinophils, thereby establishing a self-perpetuating cycle of CCL11 production and hepatic injury [8]. Recent findings by Kong et al. demonstrate the selective upregulation of CCL11 in HSCs, but not in hepatocytes or Kupffer cells, in fibrotic liver models. Furthermore, CCL11 expression in cultured HSCs is enhanced by key profibrotic mediators, specifically TGF-β and PDGF, through a mechanism involving the zinc finger factor 281 (ZNF281)-mediated transactivation of the CCL11 promoter [9].

Within the hepatocellular carcinoma (HCC) microenvironment, cancer-associated fibroblasts (CAFs) represent a predominant stromal component that is critically involved in tumor metastasis and invasion. Through comprehensive cytokine array analysis, Xu et al. revealed the significant upregulation of CCL11 along with CCL5, CCL7, CCL8, and CCL20 in CAF-conditioned medium compared to para-cancerous tissue fibroblast-derived medium [10]. The fibroblast–CCL11 axis has been further characterized in dermal models, where human dermal fibroblasts exhibit substantial CCL11 production under Th2 cytokine stimulation, while Th1 cytokines, particularly IFN-γ, exert inhibitory effects—a mechanism that potentially underlies the IFN-γ-mediated suppression of eosinophilic inflammation [11]. Watanabe et al. extended these observations by demonstrating that human dermal fibroblasts specifically produce CCL11, but not CCL24, both under basal conditions and following IL-4/IL-13 stimulation [12].

#### 1.2.3. Smooth Muscle Cells

Rahman et al. demonstrated that IL-17A stimulation triggers CCL11 secretion in human airway smooth muscle cells through the activation of multiple MAPK signaling pathways, including p38, JNK, and p42/p44 ERK, with the potential involvement of STAT-3 signaling [13]. Furthermore, CCL11 has been identified as a significant component in vascular SMCs within atherosclerotic plaques. This finding is supported by evidence showing the substantial upregulation of CCL11 mRNA levels in rat aortic SMCs following extended ischemic storage conditions [14].

#### 1.2.4. Endothelial Cells

Jaruga et al. further identified sinusoidal endothelial cells as an additional source of CCL11 production, demonstrating elevated serum CCL11 levels in a concanavalin A (ConA)-induced murine model of liver injury. This upregulation was mechanistically linked to the activation of the IL-4/STAT6 signaling pathway [15].

#### 1.2.5. Epithelial Cells

Chu et al. [16] demonstrated that bronchial epithelial cells exhibit CCL11 expression in response to IL-4 stimulation through STAT6 pathway activation, while Ma et al. [17] found that epithelial cells show inducible CCL11 mRNA expression following stimulation with pro-inflammatory cytokines, including IL-6, TNF-α, and IFN-α.

#### 1.2.6. Hepatocytes

Contrary to the findings of Kong et al. [9], who reported undetectable CCL11 levels in hepatocytes and Kupffer cells in murine liver fibrosis models, Fan et al. demonstrated that hepatocytes in non-alcoholic fatty liver disease exhibit CCL11 expression through palmitate-mediated transcriptional activation [18]. This discrepancy is further complicated by Jaruga et al.’s observation of hepatocyte-derived CCL11 expression in a ConA-induced liver injury model, mediated through the IL-4/STAT6 signaling pathway [15].

#### 1.2.7. Tumor Cells

Emerging evidence indicates that CCL11 production is not limited to stromal cells but is also exhibited by tumor cells in various malignancies, including ovarian cancer [5], hepatocellular carcinoma [19], and colorectal cancer [20]. Tumor-derived CCL11 establishes autocrine signaling loops that enhance malignant cell proliferation and invasive potential. Furthermore, tumor-secreted CCL11 exerts paracrine effects on immune cells and modulates the tumor microenvironment, collectively contributing to tumor progression.

### 1.3. CCL11-Binding Receptors

CCL11 has three receptors—CCR2, CCR3, and CCR5. The roles and mechanisms of CCL11-binding receptors are summarized in Table 2.

#### 1.3.1. Chemokine Receptor CCR3

Extensive research by Combadiere et al., along with subsequent investigations, has established CCR3 as the principal high-affinity receptor for CCL11, mediating eosinophil recruitment and activation at inflammatory sites [22,31,32,33]. Functioning as a heteromeric G-protein-coupled receptor, CCR3 initiates intracellular signaling cascades primarily through calcium mobilization [34]. Clinical studies have demonstrated a strong correlation between elevated CCR3 and ligand expression levels with disease severity in asthma patients, underscoring its therapeutic potential for eosinophil-mediated inflammatory disorders [23]. Through CCR3 engagement, CCL11 exerts potent chemotactic activity on multiple immune cell populations, including eosinophils, basophils, mast cells, TH2 lymphocytes, and dendritic cells [22,24]. Notably, CCR3 exhibits a promiscuous ligand binding capacity, recognizing a diverse array of chemokines including agonists (CCL3, 4, 5, 7, 13, 15, 23, 24, 26, and 28) and antagonists (CCL9, 10, and 18) [21].

#### 1.3.2. Chemokine Receptor CCR5 and CCR2

CCL11 demonstrates functional interactions with CCR2 and CCR5, which are two structurally homologous chemokine receptors sharing 73% sequence identity, presumably arising from gene duplication events [26,35]. While CCL11 unequivocally functions as a CCR5 agonist, its interaction with CCR2 remains controversial. Martinelli et al. proposed roles for CCL11 at CCR2, demonstrating that supraphysiological concentrations induce chemotaxis, whereas sub-stimulatory levels antagonize MCP-1-mediated CCR2 activation, suggesting partial agonist activity [27]. In contrast, Ogilvie et al. characterize CCL11 as a natural CCR2 antagonist, as is evidenced by its inability to activate CCR2-transfected cells even at micromolar concentrations, despite the robust activation of CCR5-expressing cells at nanomolar levels [25,28]. Notably, CCL11 induces CCR5 internalization in human monocytes and macrophages. Through its CCR2 antagonism, CCL11 suppresses the release of inflammatory mediators, including enzymes, leukotrienes, and histamine, thereby exhibiting anti-inflammatory properties. This dual functionality positions CCL11 as a unique modulator in chemokine-mediated inflammatory processes [28].

CCR5 demonstrates ubiquitous expression across diverse cell populations, encompassing T lymphocytes, macrophages, granulocytes, dendritic cells, microglia, astrocytes, neurons, fibroblasts, and cells within epithelial, endothelial, and vascular smooth muscle tissues. In contrast, CCR2 exhibits a more restricted expression profile, primarily localized to monocytes, natural killer cells, and T lymphocytes; however, its expression can be induced in additional cell types during inflammatory processes. While CCR2 is predominantly implicated in pro-inflammatory signaling cascades, emerging evidence suggests its involvement in anti-inflammatory mechanisms, particularly through its activity in regulatory T lymphocytes [26].

Beyond its interaction with CCL11, CCR5 exhibits high-affinity binding to multiple chemokine ligands, functioning as a receptor for agonists (CCL3, CCL4, CCL5, CCL8, CCL13, CCL14, CCL16, and CCL3L1) and the antagonist CCL7. Similarly, CCR2 demonstrates broad ligand specificity, engaging with CCL2, CCL7, CCL8, CCL12, and CCL13 in inflammatory signaling pathways [5].

#### 1.3.3. Chemokine Receptor CXCR3

CCL11 demonstrates a limited binding affinity for CXCR3, which is a chemokine receptor that is predominantly expressed on Th1 cells, suggesting its potential involvement in Th1 response modulation during pathological processes, particularly in allergic inflammation [29]. CXCR3, which is also expressed on T cells, natural killer cells, and select epithelial cell populations, serves as a receptor for multiple chemokines, including CXCL4, CXCL9, CXCL10, and CXCL11 [30].

## 2. Mechanistic Functions of CCL11 Signaling

### 2.1. The Roles of CCL11 in Relation to Immune Response

**Figure 1:**
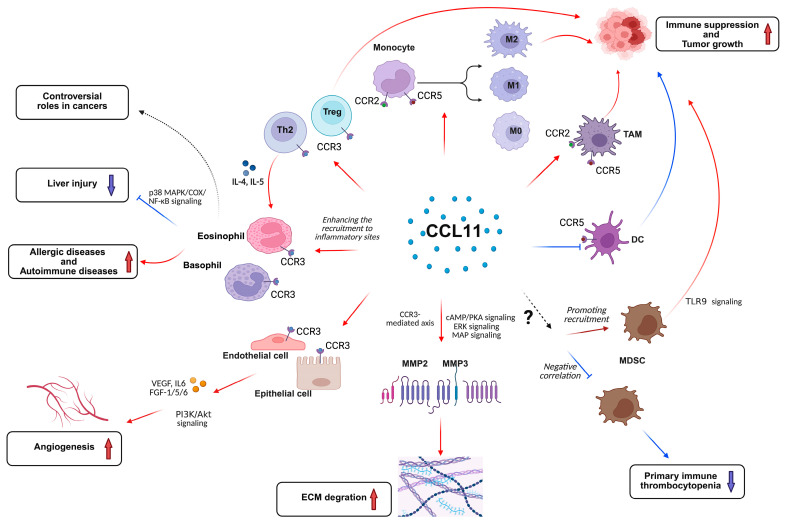
Schematic representation of CCL11 signaling mechanisms in immune regulation and disease pathogenesis. This illustration comprehensively outlines the pleiotropic functions of CCL11 in orchestrating immune cell recruitment, activation, and disease progression. Through interactions with its cognate receptors (CCR2, CCR3, and CCR5), CCL11 modulates diverse cellular responses in both immune and stromal compartments. In oncological contexts, CCL11 demonstrates dual functionality, facilitating tumor progression via immune escape mechanisms while simultaneously exhibiting tumor-suppressive properties. These effects are mediated through regulating the proliferation, differentiation, and biology of Treg, macrophage, TAM, DC, and MDSC. Furthermore, CCL11 critically regulates eosinophil and basophil biology in both inflammatory and neoplastic conditions. Beyond immune modulation, CCL11 exerts significant effects on endothelial and epithelial cell functions, while upregulating matrix metalloproteinases (MMP-2 and MMP-3) to effect angiogenesis and extracellular matrix remodeling. Red arrows represent promotion, and blue arrows represent inhibition. The figure was created by BioRender online software (BioRender.com accessed on 30 March 2025).

As a member of the CC chemokine family, CCL11 mediates the recruitment of diverse immune cell populations through receptor-dependent mechanisms, including eosinophils, basophils, T helper 2 lymphocytes, monocytes, macrophages, and myeloid-derived suppressor cells (MDSCs) [36] (Figure 1).

#### 2.1.1. Eosinophils/Basophils

Eosinophils and basophils, which undergo differentiation and maturation in the bone marrow, constitute approximately 1–6% and <1% of circulating leukocytes, respectively. These granulocytes exhibit unique migratory capabilities, transitioning from circulation to extravascular tissues upon activation—a process that is intimately associated with their involvement in various pathological conditions, including allergic disorders (asthma, allergic rhinitis, atopic dermatitis, and food allergies), autoimmune diseases, infectious processes, and malignancies [37]. CCL11 initiates multiple intracellular signaling pathways in eosinophils and basophils, promoting chemotaxis along CCL11 concentration gradients. This mechanism facilitates the targeted recruitment of these cells to inflammatory foci and prolongs their survival through autocrine and paracrine mediator release [38].

The role of CCL11 in eosinophil regulation remains controversial, with conflicting evidence from different experimental models. While some studies support its function as a key modulator of peripheral eosinophil homeostasis and tissue eosinophilia [39,40], contrasting evidence from CCL11-deficient murine models suggests that CCL11 is dispensable for airway eosinophil recruitment, potentially due to compensatory mechanisms involving alternative chemokine pathways [41]. Secondly, research has also significantly advanced the understanding of eosinophil-mediated hepatoprotection. Wang et al. first established the protective role of eosinophils in murine hepatic ischemia-reperfusion injury through ST2-dependent IL-13 secretion [42]. Building on this, Xu et al. demonstrated that IL-33-activated eosinophils orchestrate hepatic protection during acetaminophen-induced acute liver injury by inducing macrophage-derived CCL24 production, which facilitates eosinophil recruitment and suggests potential therapeutic applications [43]. Further mechanistic insights from the same research group revealed that eosinophil-derived IL-4/IL-13 production, which is regulated through the p38 MAPK/COX/NF-κB signaling axis in response to IL-33 stimulation, is crucial for hepatoprotection in acetaminophen toxicity models [44]. Thirdly, tumor-associated eosinophil infiltration may be mediated by chemoattractants that are released from necrotic neoplastic cells, with CCL11 potentially playing a significant role in this process [45,46].

#### 2.1.2. T Cells

Recent investigations have revealed that Th1 and Th2 lymphocyte subsets are selectively recruited to mediate distinct inflammatory processes. Of particular significance, CCR3—the primary receptor for CCL11, which is initially characterized on eosinophils and basophils—is also functionally expressed on Th2 cells. Through anti-CCR3 antibody-mediated isolation, researchers have successfully established Th2-polarized cell lines from peripheral blood T cells and generated in vitro-derived Th2 cells from naïve T-cell precursors. CCL11 demonstrates potent chemotactic activity in CCR3+ T cells, inducing intracellular calcium mobilization and promoting Th2-mediated immune responses through IL-4 and IL-5 production, thereby facilitating eosinophil and basophil activation in allergic inflammation [47]. Furthermore, recombinant human CCL11 has been shown to expand the CD4+CD25+Foxp3+ regulatory T-cell (Treg) population while upregulating both CCR3 and Foxp3 expression in tumor microenvironments [48].

#### 2.1.3. Monocytes/Macrophages

CCL11 exhibits significant structural homology (>60% amino acid sequence identity) with CCL2, which is a CC chemokine subfamily member that is primarily involved in monocyte and lymphocyte recruitment. This evolutionary conservation suggests a potential functional overlap in CCL11’s ability to modulate monocyte and lymphocyte activity. Experimental evidence from Xing et al. demonstrates that recombinant CCL11 induces macrophage differentiation from bone marrow precursors, generating M0-, M1-, and M2-like phenotypes through prolonged exposure (7 days) to conditioned medium from CCL11-overexpressing murine sarcoma cells (MS-Ks) [49]. Furthermore, CCL11-mediated tumor-associated macrophage (TAM) recruitment has been implicated in establishing immunosuppressive niches that facilitate tumor progression [25].

#### 2.1.4. DCs

CCL11 exerts dual immunomodulatory effects by impairing DC maturation, thereby promoting Th2 polarization and suppressing CD8+ T-cell activity, as well as differentially regulating MDSC recruitment [48,50]. While some studies show that CCL11 promotes MDSC infiltration [51], others report an inverse correlation with MDSC accumulation [52], suggesting context-dependent regulation. These mechanisms collectively contribute to an immunosuppressive tumor microenvironment.

### 2.2. The Roles of CCL11 on Angiogenesis

CCR3 expression is not restricted to leukocytes but is also present in various non-hematopoietic cell types, including human microvascular endothelial cells and airway epithelial cells [14], suggesting potential roles for CCL11/CCR3 signaling in angiogenesis. Clinical observations of elevated CCL11 levels and eosinophil infiltration in Hodgkin’s lymphoma, nasal polyposis, endometriosis, and allergic diathesis correlate with enhanced angiogenic processes, which appear to be directly mediated by CCL11 rather than being secondary to eosinophil-derived factors. Experimental evidence from Salcedo et al. demonstrates that CCL11 directly induces angiogenic responses in vivo through CCR3+ endothelial cell activation [53]. Complementary findings by Park et al. established that CCL11-CCR3 axis-mediated vascularization in human umbilical vein endothelial cells occurs via PI3K/Akt signaling pathway activation [54]. Furthermore, a microarray analysis of CCL11-stimulated human airway epithelial cells revealed the significant upregulation of multiple pro-angiogenic factors, including interleukin-6 (IL-6), fibroblast growth factors (FGF-1, FGF-5, and FGF-6), and vascular endothelial growth factors (VEGF-A and VEGF-C) [55] (Figure 1). Meanwhile, a recent study has successfully integrated tumor angiogenesis with immune cell cytotoxicity to optimize combination therapies [56]. This approach has demonstrated the roles of CCL11-mediated angiogenesis and immune modulation, providing a computational framework to predict optimal targeting strategies.

### 2.3. Matrix Metalloproteinase (MMP) Expression and Extracellular Matrix (ECM) Degradation

Emerging research has elucidated CCL11’s capacity to upregulate MMP-2 at transcriptional, translational, and functional levels in smooth muscle cells through CCR3-mediated signaling, which requires epidermal growth factor receptor activation [57]. Mechanistic studies by Chao et al. further revealed that CCL11 induces MMP-3 transcriptional activation and secretion in a concentration-dependent manner [58]. At elevated concentrations, CCL11 suppresses cAMP/protein kinase A (PKA) signaling while activating the extracellular signal-regulated kinase (ERK) and p38 mitogen-activated protein (MAP) kinase pathways to modulate MMP-3 transcription. In contrast, lower CCL11 concentrations stimulate phosphatidylinositol 3-kinase (PI3K) and c-Jun N-terminal kinase (JNK) MAP kinase pathways to enhance MMP-3 secretion—a process that is critically involved in osteoarthritis-associated cartilage degradation. These findings highlight CCL11′s central role in osteoarthritis pathogenesis and its potential utility as both a diagnostic biomarker and therapeutic target for this degenerative joint disease (Figure 1).

## 3. Roles of CCL11 Signaling in Cancers

While CCL11 has traditionally been characterized as a key mediator of eosinophil recruitment in allergic inflammation, emerging oncology research has expanded our understanding of its potential clinical applications. Recent investigations have identified CCL11 as a promising biomarker with dual utility—firstly, by detecting neoplastic processes through its association with tumor-associated inflammation, and secondly, in monitoring therapeutic responses via inflammation resolution tracking [59]. However, the complete spectrum of CCL11’s diagnostic and prognostic potential in oncology remains to be fully elucidated. This knowledge gap underscores the critical need for further research into CCL11’s multifaceted roles in tumorigenesis and cancer progression, as summarized in Table 3.

### 3.1. Pro-Tumor Functions

#### 3.1.1. Renal Cell Carcinoma (RCC)

Studies on RCC have revealed the significant overexpression of the CCL11 receptor CCR3 in tumor cells. Functional analyses demonstrate that CCL11 exerts potent mitogenic effects on RCC cells, promoting their proliferation. Clinically, CCR3 expression in tumor specimens shows a positive correlation with advanced histological grades, suggesting that CCL11/CCR3 signaling may drive tumor progression and metastatic potential in CCR3-positive RCC [60].

#### 3.1.2. Ovarian Carcinoma

Levina et al. provided compelling evidence for CCL11’s pivotal role in ovarian carcinoma progression, demonstrating its significant contribution to tumor cell proliferation and invasion through the CCR2-, CCR3-, and CCR5-mediated signaling pathways. Their findings reveal that CCL11 potently enhances malignant behaviors in ovarian carcinoma cell lines via effects that are effectively abrogated by receptor-specific neutralizing antibodies. Mechanistically, CCL11 stimulates tumor progression through the activation of key signaling phosphoproteins (ERK1/2, MEK1, and STAT3) and the upregulation of diverse molecular mediators, including cytokine receptors (IL-6R), chemokines (MIF, CXCL8, G-CSF, GM-CSF, and M-CSF), growth/angiogenic factors (VEGF and SDF-1α), and adhesion molecules (ICAM-1). Clinically, ovarian cancer patients exhibit significantly reduced serum CCL11 levels compared to healthy controls and patients with other malignancies, with these levels inversely correlating with relapse-free survival post resection. Importantly, therapeutic intervention through CCL11 axis blockade using neutralizing antibodies significantly enhances cisplatin sensitivity in ovarian carcinoma cells, suggesting promising combination therapy strategies [5].

#### 3.1.3. Breast Cancer

In breast cancer patients, elevated serum CCL11 levels correlate with the expansion of CD4+CD25+Foxp3+Tregs and the enhanced production of IL-2 and TGF-β1 through STAT5 signaling pathway activation. Considering STAT5’s critical role in immune homeostasis maintenance—particularly through its support of Treg cell development and function, as well as the direct regulation of Foxp3 transcription via binding to regulatory genomic elements—the pharmacological modulation of this pathway represents a promising therapeutic strategy for cancer immunotherapy [48]. Furthermore, the CCL11/CCR3 axis has been implicated in facilitating breast cancer lung metastasis under conditions of asthma-associated chronic inflammation, suggesting a potential link between allergic inflammation and cancer progression [61].

#### 3.1.4. Pancreatic Ductal Adenocarcinoma

TLR9 activation in pancreatic ductal adenocarcinoma exerts a pro-tumorigenic influence on epithelial cells by mediating the recruitment of MDSCs through the release of CCL11 [51].

#### 3.1.5. Lymphoma

Miyagaki et al. revealed that CCL11/CCR3 interactions could enhance the tumor progression of anaplastic large cell lymphoma in vitro/vivo via activating ERK1/2 signaling [62].

### 3.2. Anti-Tumor Functions

#### Fibrosarcoma

Xing et al. identified distinct CCL11 expression patterns across murine fibrosarcoma cell lines, revealing an inverse correlation between CCL11 levels and angiogenic potential. The NFSA cell line, which is characterized by a high CCL11 expression, exhibited restricted angiogenesis and extensive necrosis, while the MS-K line with minimal CCL11 expression demonstrated robust tumor growth and well-developed vascular networks. These findings suggest that CCL11-mediated eosinophil recruitment may exert anti-angiogenic effects and promote necrotic processes in fibrosarcoma progression [49].

### 3.3. Dual or Controversial Roles in Cancers

#### 3.3.1. Non-Small-Cell Lung Cancer (NSCLC)

Lin et al. elucidated a novel metastatic mechanism in NSCLC, demonstrating that MDSC-secreted CCL11 promotes tumor progression through ERK/AKT pathway activation and epithelial–mesenchymal transition (EMT) induction. This MDSC-CCL11-ERK/AKT-EMT axis represents a promising therapeutic target for NSCLC metastasis inhibition [63]. Contrastingly, Siva et al. observed differential CCL11 dynamics in NSCLC patients, with chemoradiotherapy resulting in a more pronounced CCL11 reduction compared to radiotherapy alone, followed by gradual post-treatment recovery—a pattern reflecting treatment efficacy against CCL11-producing cells [64]. Furthermore, Tsao et al. established the prognostic significance of CCL11 in NSCLC, revealing that lower pretreatment serum CCL11 levels correlate with reduced progression-free survival in vandetanib-treated patients, suggesting its potential as a predictive biomarker [65].

#### 3.3.2. Colorectal Cancer (CRC)

Emerging evidence demonstrates elevated CCL11 expression levels in CRC tissues compared to normal controls, suggesting its involvement in CRC pathogenesis [66]. Polosukhina et al. further highlighted the critical role of epithelial-derived CCL11 in modulating colitis-associated carcinogenesis, proposing anti-CCL11 antibody therapy as a promising strategy for both inflammatory bowel disease management and cancer chemoprevention [67]. Paradoxically, CRC patients exhibit significantly reduced plasma CCL11 levels compared to healthy individuals, with the degree of reduction correlating with advanced Duke’s staging [66]. Cho et al. revealed complex CCL11 expression patterns in colorectal neoplasms, characterized by decreased glandular expression but increased stromal expression, which is a spatial distribution pattern that may promote tumor immune evasion by attenuating eosinophil-mediated antitumor responses [20]. However, the precise mechanistic implications of glandular CCL11 downregulation in CRC immune escape and progression remain to be fully elucidated.

#### 3.3.3. Prostate Cancer

Zhu et al. demonstrated that CCL11 promotes prostate cancer cell migration and invasion through CCR3-mediated ERK pathway activation and subsequent MMP-3 upregulation [68]. Their mechanistic studies revealed that CCR3 silencing effectively inhibits these malignant behaviors by suppressing ERK1/2 phosphorylation and MMP-3 expression. Consistent with these findings, the pharmacological inhibition of the ERK pathway attenuates CCL11-induced tumor cell invasion and migration while reducing MMP-3 production. Complementing these in vitro findings, Heidegger et al. observed significantly decreased serum CCL11 levels in both prostate cancer and benign prostatic hyperplasia patients compared to healthy controls. Although the prognostic significance of CCL11 in prostate cancer remains undefined, these findings suggest its potential utility as a diagnostic biomarker for distinguishing between normal and pathological prostate conditions [69].

#### 3.3.4. Esophageal Cancer

Blank et al. revealed a complex prognostic relationship between CCL11 expression patterns and esophageal cancer outcomes. Their findings demonstrate that reduced intratumoral CCL11 levels correlate with favorable prognosis, while decreased serum CCL11 concentrations are associated with poorer clinical outcomes. This paradoxical tissue–serum dichotomy likely reflects distinct regulatory mechanisms, with CCL11 expression in these compartments being independently modulated by diverse immunogenic factors [70].

#### 3.3.5. Melanoma

Simson et al. revealed that a combination of IL5 and CCL11 could successfully recruit eosinophils into a melanoma, which causes immune evasion and tumor eradication [71].

#### 3.3.6. Hepatocellular Carcinoma

In HCC, similar to observations in melanoma, CCL11-expressing tumors demonstrate that IL-5/CCL11 co-stimulation effectively recruits tumor-associated eosinophils, potentially mediating both immune evasion and tumoricidal effects [19]. Clinical investigations by Ng et al. involving 150 liver transplant recipients revealed that elevated post-transplant circulating CCL11 levels significantly correlate with HCC recurrence, suggesting its potential as a recurrence biomarker [72]. Nevertheless, the precise mechanisms and individual contributions of CCL11 in HCC remain poorly understood, compounded by the dualistic nature of eosinophil and other immune cell functions within tumor microenvironments.

## 4. Roles of CCL11 Signaling in Liver Diseases

### 4.1. Liver Injury

Jaruga et al. established a ConA-induced T-cell-mediated murine liver injury model, demonstrating that upregulated CCL11 expression in hepatocytes and sinusoidal endothelial cells, accompanied by elevated serum levels, drives eosinophil and neutrophil recruitment to injured hepatic tissue [15]. Their therapeutic intervention studies revealed that CCL11 blockade significantly attenuates hepatitis severity and reduces leukocyte infiltration [73]. Clinical observations further support these findings, with elevated CCL11 levels being detected in drug-induced liver injury patients, where it facilitates hepatic eosinophil recruitment [74]. Despite these insights, comprehensive clinical data regarding CCL11 expression patterns across various liver diseases remain limited, highlighting the need for further systematic investigation.

A recent study has demonstrated that liver injury (such as partial hepatectomy or toxicant-induced injury) can activate some signaling pathways that maintain essential hepatic functions (including synthesis, metabolism, and detoxification), while simultaneously initiating tissue repair through cellular replication [75]. Liver regeneration represents a complex but vital physiological response to parenchymal loss, ultimately restoring hepatic architecture and functionality [76]. The dual role of CCL11 in both injury response and regeneration provides critical insights into liver pathophysiology and liver diseases. Fan et al. demonstrated the significant upregulation of CCL11 expression in regenerating murine livers compared to quiescent hepatic tissue, suggesting its involvement in liver regeneration processes. Their mechanistic studies revealed that Brg1, which is a chromatin remodeling factor, regulates eosinophil recruitment through CCL11 transcriptional activation, thereby supporting hepatic regeneration. Furthermore, hepatocyte growth factor stimulation induces CCL11 expression in primary murine hepatocytes. At the molecular level, Brg1 facilitates CCL11 transcription through direct interaction with NF-κB/RelA, highlighting its central role in orchestrating liver regeneration dynamics [77].

### 4.2. Liver Cirrhosis

Kong et al. identified a novel profibrotic function of CCL11 in HSC-mediated liver fibrogenesis using murine models, providing mechanistic insights that support the therapeutic potential of CCL11-targeted interventions [9]. Complementing these findings, Tacke et al. established the clinical relevance of CCL11 as a plasma biomarker, demonstrating that elevated levels correlate with hepatic inflammation severity, advanced fibrosis stages, and poor prognosis in liver cirrhosis patients. These collective findings position CCL11 as both a diagnostic marker for histological fibrosis assessment and a prognostic indicator for clinical outcomes in chronic liver disease [73].

### 4.3. Hepatitis and Cholangitis

In the context of viral hepatitis, Wong et al. elucidated a critical interplay between CCL11 and IL-13 in chronic hepatitis B virus infection, demonstrating that these mediators integrate metabolic and inflammatory pathways that independently drive liver cirrhosis progression [78]. Expanding the spectrum of liver diseases, Landi et al. conducted a comprehensive chemokine/cytokine profiling study in primary sclerosing cholangitis (PSC), primary biliary cholangitis (PBC), and autoimmune hepatitis (AIH) patients, with chronic hepatitis C and healthy controls as comparators. Their findings revealed disease-specific CCL11 expression patterns, whereby elevated levels in PSC, typically diagnosed at advanced fibrotic stages, contrasted with reduced levels in PBC and AIH [8].

### 4.4. Alcoholic Liver Disease (ALD) and Non-Alcoholic Fatty Liver Disease (NAFLD)

Li et al. established a crucial role for CCL11 in ALD pathogenesis, demonstrating that ethanol exposure upregulates hepatocyte CCL11 expression in both in vivo and in vitro models. Their findings reveal that the CCL11/CCR3 axis drives macrophage-mediated inflammation and hepatic steatosis, while the genetic ablation of CCL11 or a pharmacological CCR3 blockade significantly attenuates ALD progression [79]. Parallel investigations by Fan et al. identified CCL11 as a novel regulator in NAFLD pathogenesis, demonstrating that CCL11 neutralization or receptor antagonism ameliorates disease progression in knockout mouse models. Clinical correlations further support these findings, showing positive associations between CCL11 expression levels and NAFLD severity in human patients [18]. Moreover, in obese NAFLD patients, CCL11 contributes to atherosclerosis development and insulin resistance through complex interactions with inflammatory and immune mediators [80].

## 5. Potential Treatments Targeting CCL11 and Its Receptors

### 5.1. CCL11

Bertilimumab (CAT-213), which is a humanized anti-CCL11 monoclonal antibody developed through phage-display technology, was originally designed for allergic disease management. In cancers, the pharmacological inhibition of CCL11 signaling has been shown to enhance chemosensitivity, as demonstrated by an improved response to cisplatin in ovarian carcinoma models [5]. Additionally, targeting the CCL11 pathway represents a promising therapeutic strategy for suppressing metastatic progression in NSCLC [63]. In liver diseases, the neutralization of CCL11 has been reported to attenuate Con A-induced hepatic injury by reducing leukocyte infiltration [15]. Furthermore, the therapeutic blockade of the CCL11/CCR3 axis has been shown to ameliorate liver fibrosis in preclinical murine models [9]. The deletion of CCL11 or pharmacological CCR3 inhibition could also significantly attenuate the progression of ALD [79]. Similarly, CCL11/CCR3 antagonism has been demonstrated to mitigate disease severity in NAFLD mouse models, suggesting its potential as a therapeutic target for metabolic liver disorders [18]. Furthermore, studies have also demonstrated its efficacy in reducing tissue eosinophil infiltration across various in vivo models, prompting its current clinical evaluation for asthma treatment [81]. Local administration studies in murine models have further confirmed CAT-213’s ability to suppress CCL11-induced dermal eosinophilia [82]. Following successful Phase II trials for nasal congestion relief, bertilimumab is currently being investigated for ulcerative colitis treatment in a clinical trial led by Eran Goldin (ClinicalTrials.gov identifier: NCT01671956) [22]. Concurrently, the development of additional anti-CCL11 monoclonal antibodies with therapeutic potential continues to progress.

### 5.2. CCR3

The development of CCR3-targeted therapeutics has yielded numerous small-molecule inhibitors and antagonists, with SB-328437 and F-1322 emerging as particularly promising candidates. SB-328437, which is a frontrunner for clinical translation, demonstrates the potent inhibition of CCL11-mediated eosinophil chemotaxis. Meanwhile, F-1322 exhibits the dose-dependent suppression of airway eosinophil infiltration in guinea pig models [83]. Complementing these small molecules, the CCR3-specific monoclonal antibody 7B11 has shown significant efficacy in vitro, highlighting its potential as a therapeutic agent for allergic disorders [84]. Furthermore, preclinical studies using chronic asthma models have validated the therapeutic potential of small-molecule CCR3 antagonists in reducing airway eosinophilia, supporting their continued development for allergic disease management. In cancers, the pharmacological inhibition of CCR3 has emerged as a promising therapeutic strategy. For instance, the CCR3-specific antagonist SB328437 significantly suppresses peritoneal metastasis in ovarian carcinoma by disrupting tumor–stromal interactions [85]. Similarly, in melanoma models, CCR3 blockade has been shown to inhibit angiogenesis, thereby impairing tumor vascularization and metastatic potential [85]. In liver diseases, targeting CCR3 has demonstrated efficacy in ameliorating a spectrum of hepatic pathologies. A recent study has indicated that anti-CCR3 therapy can attenuate liver fibrosis by reducing the activation of HSCs and collagen deposition [9]. Furthermore, the inhibition of the CCL11/CCR3 signaling axis ameliorates ALD by influencing hepatic microenvironment and hepatocyte injury [79]. In NAFLD, CCR3 antagonism mitigates disease progression by modulating lipid metabolism and reducing hepatic steatosis [18].

### 5.3. CCR5

The therapeutic potential of chemokine receptor targeting was first realized through their identification as crucial HIV co-receptors, with CCR5 and CXCR4 playing pivotal roles in viral entry. Notably, CCR5 mediates the majority of global HIV transmissions, underscoring its clinical significance. This discovery led to the development of maraviroc, the first FDA-approved chemokine receptor-targeting drug. Building on this success, numerous CCR5-directed therapeutic strategies have emerged, including monoclonal antibodies and small molecule inhibitors that are currently undergoing clinical evaluation. Prominent candidates in this class include GSK706769, INCB009471, vicriviroc, and GW873140, all demonstrating potential for HIV infection management [86,87].

In oncological therapeutics, the pharmacological targeting of CCR5 has exhibited considerable promise across diverse malignant neoplasms. The CCR5 antagonist maraviroc, which was initially developed as an antiretroviral agent for HIV management, has been successfully repurposed for metastatic breast cancer intervention. Mechanistic studies demonstrate its efficacy in attenuating pulmonary metastatic dissemination through the selective inhibition of CCR5-mediated tumor cell migration and metastatic niche colonization [88]. Similarly, in prostate cancer, the humanized monoclonal CCR5 inhibitor has shown promising anti-tumor effects by impairing CCR5-dependent tumor proliferation and modulating the tumor microenvironment [88]. The clinical translation of these findings is underway, as evidenced by an ongoing Phase I/II trial (NCT02704962) investigating maraviroc combined with conventional chemotherapy in colorectal cancer patients, with preliminary data confirming acceptable safety profiles and hinting at potential efficacy [88]. In hepatic pathology, the CCR5 blockade has emerged as a viable strategy for managing fibrotic progression. Notably, maraviroc treatment in HIV/HCV co-infected patients significantly improved liver fibrosis parameters, suggesting CCR5’s crucial role in fibrogenesis through its regulation of hepatic stellate cell activation and pro-fibrotic cytokine production. These clinical observations are supported by preclinical studies demonstrating that CCR5 deficiency attenuates liver fibrosis in experimental models [89]. The dual utility of CCR5 inhibitors in both oncological and hepatological contexts underscores the pleiotropic nature of chemokine signaling in disease pathogenesis, highlighting the potential for drug repurposing strategies.

### 5.4. CCR2

The CCL11 receptor CCR2 has emerged as a critical therapeutic target in multiple disease pathways, primarily through its interaction with CCL2. Current drug development efforts focus on inflammatory and metabolic disorders, including atherosclerosis and rheumatoid arthritis, utilizing MLN1202, which is a humanized anti-CCR2 monoclonal antibody that blocks CCL2 binding and downstream signaling. Clinical research has progressed to Phase II trials with BMS-741672, which is a small molecule CCR2 antagonist that is being evaluated for type 2 diabetes management. The parallel preclinical development of indolopiperidine-derived CCR2 inhibitors further highlights the therapeutic promise of CCR2 modulation in treating complex inflammatory and metabolic diseases [90,91].

Emerging evidence highlights CCR2 as a promising therapeutic target in both oncological and hepatological contexts. In esophageal cancer, the pharmacological blockade of CCR2 signaling significantly reduces tumor incidence by impairing the recruitment of TAMs, which subsequently enhances the antitumor activity of CD8+ T cells within the tumor microenvironment [92]. This immunomodulatory approach demonstrates particular promise in pancreatic cancer, where CCR2 inhibitors synergize with conventional chemotherapy to reduce TAM infiltration and restore antitumor immunity [85]. Similarly, in HCC models, the specific CCR2 antagonist RS504393 exhibits potent anti-angiogenic effects, effectively suppressing tumor neovascularization and growth [85]. The therapeutic potential of CCR2 inhibition extends to metabolic liver diseases, where clinical studies have demonstrated significant antifibrotic effects. In patients with NAFLD and steatohepatitis, the CCR2 antagonist CCX872-B has shown clinical efficacy in reducing fibrosis scores, as evidenced by improved histopathological parameters in phase II clinical trials [93,94]. The dual efficacy of CCR2 inhibitors in both cancer and liver disease models underscores the fundamental role of this chemokine pathway in regulating tissue fibrosis and tumor progression, highlighting its potential as a therapeutic target across multiple disease states.

Thus, potential treatments targeting CCL11 and its receptors are summarized in Table 4.

## 6. Conclusions and Future Perspectives

This review highlights the dualistic roles of CCL11 in cancer and liver diseases, emphasizing its context-dependent functions as both a tumor-promoting and tumor-suppressing chemokine. In cancers such as renal cell carcinoma, ovarian carcinoma, and breast cancer, CCL11 drives tumor progression through immune evasion, angiogenesis, and extracellular matrix remodeling. Conversely, in fibrosarcoma, it exhibits tumor-suppressive effects by inhibiting angiogenesis and promoting necrosis. Its complex roles in non-small-cell lung cancer, colorectal cancer, and hepatocellular carcinoma further underscore its multifaceted involvement in tumorigenesis. In liver diseases, CCL11 plays critical roles in liver injury, cirrhosis, hepatitis, and hepatic repair. Its involvement in ALD and NAFLD highlights its relevance in metabolic and inflammatory pathways, making it a promising biomarker and therapeutic target. The therapeutic targeting of CCL11 and its receptors, including monoclonal antibodies (e.g., bertilimumab) and small-molecule inhibitors (e.g., maraviroc), shows significant preclinical promise.

However, critical research gaps remain, particularly in understanding the tissue-specific mechanisms underlying CCL11’s paradoxical roles in different cancer types; how CCL11 crosstalk with other chemokines (e.g., CCL2/CCL5) modulates disease progression in liver pathologies; and the molecular determinants of resistance to CCL11-targeted therapies. Additionally, the lack of clinical data on CCL11 inhibition in advanced liver diseases represents a major translational hurdle. Addressing these challenges—including off-target effects and optimal therapeutic windows—will be essential for clinical optimization.

Future studies should leverage advanced technologies (e.g., single-cell RNA sequencing and spatial transcriptomics) to dissect CCL11’s spatiotemporal actions in tumor and hepatic microenvironments. Large-scale clinical trials are urgently needed to validate CCL11 as a stratified biomarker and evaluate combination therapies (e.g., CCL11 inhibitors with immune checkpoint blockades). By bridging these mechanistic and translational gaps, CCL11-targeted strategies could unlock novel paradigms in precision oncology and hepatology, ultimately improving patient outcomes.

## Figures and Tables

**Table 1:** Summary of CCL11 production.

Cell Types		Diseases	Stimulating Factors	Ref.
Eosinophils		Asthma, ulcerative colitis, Crohn’s disease, drug-induced liver injury	Th2 cytokines, like IL4	[6,7]
Fibroblasts	HSCs	Liver fibrosis	(1) Pro-fibrogenic growth factors: TGF-β, PDHF (2) Promoter: zinc finger factor 281	[8,9]
	CAFs	Metastasis and invasion of HCC	Under stimulations of inflammatory cytokines	[10]
	Dermal fibroblasts	Cutaneous inflammation	(1) Promoted by IL4/13 (2) Inhibited by IFN-γ	[11,12]
Smooth muscle cell	Airway smooth muscle cell	Allergic asthma	(1) Under IL-17A stimulation (2) Signaling: MAPK, STAT-3 pathway	[13]
	Vascular smooth muscle cells	Atherosclerosis	Ischemic situation	[14]
Endothelial cells		ConA-induced liver injury	IL-4/STAT6 signaling pathway	[15]
Epithelial cells		Allergic asthma	(1) IL-4/STAT6 signaling pathway (2) With the stimulations of TNF-a, IL-1, or IFN-a	[16,17]
Hepatocytes		NASH, ConA-induced liver injury	(1) Activated by free fatty acids (2) IL-4/STAT6 signaling pathway	[15,18]
Tumor cells		Ovarian cancer, HCC, colorectal cancer	Under stimulations of inflammatory cytokines	[5,19,20]

**Table 2:** Summary of CCL11 receptors—CCR3, CCR5, CCR2, and CXCR3.

CCL11 Receptor	Key Roles	Major Expressing Cells	Other Binding Chemokines	Ref.
CCR3	Recruits eosinophils and basophils	Eosinophils, basophils, TH2 lymphocytes, mast cells, and dendritic cells	(1) Agonist: CCL 3, 4, 5, 7, 13, 15, 23, 24, 26, 28 (2) Antagonist: CCL 9, 10, 18	[21,22,23,24]
CCR5	Induces the internalization of CCR5 in monocytes and macrophages	T lymphocytes, macrophages, dendritic cells (DCs), granulocytes, fibroblasts, epithelium, endothelium, and vascular smooth muscle	(1) Agonist: CCL3, CCL4, CCL5, CCL8, CCL13, CCL14, CCL16, CCL3L1 (2) Antagonist: CCL7	[5,25,26]
CCR2	Dual activity of CCL11/CCR2 on inflammatory effects by partial agonists or natural antagonists in different studies	Monocytes, NK, and T lymphocytes	(1) Agonist: CCL2, CCL7, CCL8, CCL12, CCL13	[26,27,28]
CXCR3	(1) Low affinity for binding CXCR3 (2) Impaired Th-1 response	T cells, NK cells, and some epithelial cells	(1) Agonist: CXCL4, CXCL9, CXCL10, CXCL11	[29,30]

**Table 3:** The functions of CCL11 on different types of cancers.

Functions	Cancer Types	Mechanisms of CCL11 Signaling	Ref.
Pro-tumor	Renal cell carcinoma	(1) Stimulate progression and invasiveness of CCR3+ RCC. (2) Correlation: overexpression of CCR3 with the grade of malignancy.	[60]
	Ovarian carcinoma	(1) Promote the proliferation and dissemination of ovarian carcinoma cells by CCR2/3/5. (2) The growth-stimulatory effects of CCL11 on activation of ERK1/2, MEK1, and pSTAT3, as well as increased levels of CXCL8, G-CSF, GM-CSF, M-CSF, IL6R, IL8, VEGF, SDF-1a, and ICAM-1. (3) Biomarker: decreased circulating levels of CCL11 in cancer. (4) Prognostic: postoperative levels of CCL11 were negatively correlated with relapse-free survival. (5) Therapy: inhibition of CCL11 signaling increased sensitivity to cisplatin.	[5]
	Breast cancer	(1) Overexpression of serum CCL11 increased the proportion of Tregs and the production of TGF-β1 and IL-2 through the STAT5 signaling pathway. (2) Promote the lung metastasis of breast cancer via CCL11.	[48,61]
	Pancreatic ductal adenocarcinoma	Protumorigenic effects on TLR9/CCL11/MDSC recruitment.	[51]
	Lymphoma	Enhance tumor growth via CCL11/CCR3/ERK1/2 signaling.	[62]
Anti-tumor	Fibrosarcoma	Reduce the formation of blood vessels and induce tumor necrosis by CCL11-induced eosinophils.	[49]
Dual/ controversial roles	Non-small-cell lung cancer	Pro-tumor: Promote tumor metastasis via MDSC-CCL11-ERK/AKT-EMT axis. Anti-tumor: (1) High ability to destroy cancer cells. (2) Low serum concentrations of CCL11 were associated with poor prognosis after vandetanib treatment.	[63,64,65]
	Colorectal cancer	Pro-tumor: (1) Higher levels of tissue CCL11 were found in CRC patients. (2) Epithelial-cell-derived CCL11 was associated with carcinogenesis. Anti-tumor: (1) CCL11 serum levels were significantly below that of controls. (2) Decreased expression of CCL11 in tumor glandular cells induced a decrease in eosinophilia and achieved immune evasion.	[20,66,67]
	Prostate cancer	Pro-tumor: Promote cancer cell migration and invasion by CCR3/ERK1/2/MMP3. Anti-tumor: Prognostic: decreased serum levels of CCL11 in cancer and hyperplasia compared to normal.	[68,69]
	Esophageal cancer	Pro-tumor: Lower levels of serum CCL11 were associated with an adverse prognosis. Anti-tumor: Lower levels of CCL11 in tissue and serum were correlated with better survival.	[70]
	Hepatocellular carcinoma	(1) A combination of IL5 and CCL11 could recruit eosinophils into the tumors and cause immune evasion. (2) The single roles of CCL11 on HCC are unclear.	[19]
	Melanoma	(1) A combination of IL5 and CCL11 could recruit eosinophils into the tumors and cause immune evasion. (2) The single roles of CCL11 on melanoma are unclear.	[71]

**Table 4:** Drugs targeting CCL11 and its receptors.

Target	Drug Names	Targeting Diseases			Ref.
		Cancers	Liver Diseases	Other Diseases	
CCL11	Bertilimumab	Ovarian carcinoma, NSCLC	Liver injury, liver fibrosis, ALD, NAFLD	Allergic disorders, asthma, dermal eosinophilia, nasal congestion, ulcerative colitis	[5,9,15,18,36,63,79,81,82,95]
CCR3	SB-328437, F-1322, 7B11	Ovarian carcinoma, melanoma	Liver fibrosis, ALD, NAFLD	Allergic diseases, chronic asthma	[9,18,79,83,84,85]
CCR5	Maraviroc, GSK706769, INCB009471, vicriviroc, GW873140	Breast cancer, prostate cancer, colorectal cancer	Liver fibrosis	HIV-1	[86,87,88,89]
CCR2	MLN1202, BMS-741672 indolopiperidine derivative	Esophageal cancer, pancreatic cancer, HCC	NAFLD, steatohepatitis	Allergic diseases, atherosclerosis, rheumatoid arthritis, type II diabetes	[85,90,92,93,94]

## Abbreviations

The following abbreviations are used in this manuscript:

CCL11	C-C motif ligand 11
MCP	Macrophage chemoattractant protein
HSC	Hepatic stellate cell
HCC	Hepatocellular carcinoma
CAFs	Cancer-associated fibroblasts
SMCs	Smooth muscle cells
ConA	Concanavalin A
MDSCs	Myeloid-derived suppressor cells
Treg	Regulatory T cell
MS-K	Murine sarcoma cell line
TAM	Tumor-associated macrophage
IL-6	Interleukin-6
FGF	Fibroblast growth factors
VEGF	Vascular endothelial growth factor
MMP	Matrix Metalloproteinase
ECM	Extracellular matrix
PKA	Protein kinase A
ERK	Extracellular signal-regulated kinase
MAP	Mitogen-activated protein
PI3K	Phosphatidylinositol 3-kinase
JNK	c-Jun N-terminal kinase
RCC	Renal cell carcinoma
NSCLC	Non-small-cell lung cancer
EMT	Epithelial–mesenchymal transition
CRC	Colorectal cancer
HSCs	Hepatic stellate cells
PSC	Primary sclerosing cholangitis
PBC	Primary biliary cirrhosis
AIH	Autoimmune hepatitis
ALD	Alcoholic liver disease
NAFLD	Nonalcoholic fatty liver disease
